# Investigation of Exposure to Plasticizers Among Electronic Waste Recycling Workers and Their Health: Protocol for a Cross-Sectional Study

**DOI:** 10.2196/96702

**Published:** 2026-08-18

**Authors:** Rahel Mesfin Ketema, Thanh Hai Le, Son Van Nguyen, Hien Thi Thu Ngo, Yi Zeng, Ayaka Yasuda, Megasari Marsela, Machiko Minatoya, Thai Ha Le, Binh Thi Ta, Van Thi Pham, Huyen Thi Nguyen, Tuan Van Pham, Hoai Son Tran, Phuong Lan Ha, Chinh Thi Thuy Phan, Yu Ait Bamai, Reiko Kishi, Atsuko Ikeda

**Affiliations:** 1 Center for Environmental and Health Sciences Hokkaido University Sapporo, Hokkaido Japan; 2 Faculty of Health Sciences Hokkaido University Sapporo, Hokkaido Japan; 3 WHO Collaborating Centre for Environmental Health and Prevention of Chemical Hazards (JPN-91) Sapporo, Hokkaido Japan; 4 National Institute of Occupational and Environmental Health, Vietnam Hanoi Vietnam; 5 WHO Collaborating Centre for Occupational Health (VTN-3) Hanoi Vietnam; 6 Faculty of Public Health, Phenikaa School of Medicine and Pharmacy, Phenikaa University, Duong Noi Ward Hanoi City Vietnam; 7 School of Public Health Jiangxi Medical College Nanchang University Nanchang, Jiangxi China; 8 Graduate School of Pharmaceutical Sciences, Health Sciences University of Hokkaido Sapporo Japan; 9 Viet Nam Administration of Disease Prevention Ministry of Health, Vietnam Hanoi Vietnam; 10 One Health Research Center Hokkaido University Sapporo, Hokkaido Japan

**Keywords:** electronic waste, occupational health, cross-sectional, phthalates, e-waste recycling village, Vietnam

## Abstract

**Background:**

The global consumption of electronic and electrical products has increased the generation of electronic waste (e-waste), which poses environmental and public health concerns. In Vietnam, informal e-waste processing, characterized by unregulated collection and dismantling, is widespread and exposes workers to hazardous substances. However, data on the health status of e-waste workers remain limited, and exposure to phthalates, which are endocrine-disrupting chemicals widely used in electronics, has not yet been evaluated.

**Objective:**

This study aims to assess the health status of e-waste workers in Vietnam, measure their phthalate exposure levels, and examine associations with health outcomes.

**Methods:**

We conducted a cross-sectional study of adult e-waste workers in Bui village, Vietnam. Trained staff collected demographic, occupational, respiratory, and dietary habit information using a structured questionnaire in Vietnamese. Medical examinations were performed and body composition measurements, blood samples, and urine samples were obtained. Blood samples were analyzed for hematological parameters and several biochemical markers; urine samples were collected for routine urinalysis parameters and phthalate quantification.

**Results:**

Field survey data were collected in March 2025, and 253 e-waste workers were recruited. Preliminary descriptive analysis indicated that most participants were female, had low income, and relied on e-waste processing as their primary income. Personal protective equipment, particularly dust masks and gloves, was commonly used. Laboratory measurements of urinary phthalate metabolites were completed by November 2025.

**Conclusions:**

This protocol provides a framework for evaluating the health status, metabolic indicators, and phthalate exposure among e-waste workers in Vietnam and supports occupational health evaluation. The preliminary findings highlight the feasibility of this study, which supports the evaluation of occupational exposure and health outcomes.

**International Registered Report Identifier (IRRID):**

DERR1-10.2196/96702

## Introduction

### Background

As digital technologies advance in modern life, the global consumption of electronic products increases, leading to a significantly more electronic waste (e-waste) generation. Improper management or inadequate recycling of e-waste can release toxic chemicals such as persistent organic pollutants (POPs), phthalates, and heavy metals into the environment [1-3]. Exposure to these chemicals has been associated with cardiovascular disease (CVD) [4], kidney disorders [5], endocrine disruption, and respiratory and allergic conditions [6]. Improper e-waste handling practices include unsafe collection, manual dismantling without proper tools, open burning, and informal recycling. These activities, together with the inadequate use of personal protective equipment (PPE) such as gloves, masks, and safety goggles, can increase not only exposure to hazardous chemicals but also the risk of physical health problems such as injuries, headaches, back pain, and blurred vision in recyclers [7,8]. These growing concerns have prompted research on the environmental and health impacts of e-waste recycling, particularly in countries where informal recycling is prevalent and regulatory enforcement is limited [2].

### Country Context

In Vietnam, informal e-waste processing areas are primarily concentrated in the northern part of the country and have been focal points for environmental and health studies. Informal e-waste processing or recycling is characterized as unregistered with the local authorities, unregulated, lacking structure, and illegal [9]. The lack of formal e-waste processing in Vietnam has exacerbated environmental and human health issues owing to exposure to toxic chemicals. Studies have shown that higher levels of environmental contamination by POPs and heavy metals are found in e-waste processing areas than in reference sites without e-waste processing activities in Vietnam [10,11]. These POPs include legacy flame retardants such as polybrominated diphenyl ethers and polychlorinated biphenyls, as well as heavy metals such as lead, cadmium, nickel, chromium, and arsenic that have been associated with respiratory conditions, hormonal or endocrine disruption, oxidative stress, DNA damage, CVDs, nervous system alteration, and altered immunological function in e-waste processing areas [12-14].

However, our narrative review on e-waste in Vietnam highlights the following research gaps: while previous studies have explored exposure to POPs and heavy metals in e-waste processing environments, general health assessment studies among workers remain limited, and no research has addressed exposure to short half-life chemicals such as phthalates [15]. Phthalates are synthetic chemicals that are used as plasticizers in electronics and plastics. They can easily leach from discarded products into the environment and have been found in e-waste recycling areas, thereby increasing the exposure levels for workers [1,16]. Recent findings from the European Human Biomonitoring Initiative have confirmed the presence of phthalates and their replacements, such as 1,2-cyclohexane dicarboxylic acid di-isononyl ester, in both e-waste dismantling workers and indoor dust collected from recycling facilities across Europe [1]. Several phthalates, including dibutyl phthalate (DBP), butyl benzyl phthalate (BBzP), di-(2-ethylhexyl) phthalate (DEHP), and diisononyl phthalate (DINP), have been classified as reproductive toxicants by regulatory authorities such as the European Chemicals Agency [17]. Studies in the general population have shown that exposure to phthalates is associated with endocrine disruption, reproductive and developmental effects, CVDs, obesity, kidney disease, and respiratory symptoms [18-21]. Furthermore, inadequate PPE use during e-waste processing may be associated with respiratory conditions, such as wheezing, rhinoconjunctivitis, and eczema, as well as subjective symptoms, which may result from the e-waste processing environment or increased phthalate exposure due to the work environment [8,22].

In addition to chemical exposure, structural inequalities in Vietnam’s occupational health system further disadvantage workers in the informal sector. For formal workers, employers are responsible for organizing occupational health checkups and covering the costs of medical examinations, diagnoses, and treatments of occupational diseases. This is included in clause 2, article 7 of law number 84/2015/QH13 on occupational safety and hygiene on the obligations of employers and clauses 1 and 6 of the law on the rights of employees working under labor contracts [23]. However, informal workers must either ensure their own health insurance or bear the full cost of health checkups. Consequently, many avoid annual checkups and seek care only when they become ill. The lack of such health care access further heightens the vulnerability to chemical exposure and work-related health conditions.

Given the abovementioned challenges and research gaps, there is an urgent need to assess the health status of informal e-waste workers in Vietnam, with a focus on short half-life plasticizers, such as phthalates.

### Objectives of This Study

This study aimed to describe the general health conditions of e-waste workers, including physical health conditions such as injuries, headaches, back pain, and blurred vision, as well as the prevalence of high blood pressure, subjective symptoms of asthma, and allergies. It also aimed to identify factors associated with the health status of workers living in an e-waste processing village in northern Vietnam. Additionally, the study assessed the exposure levels of short half-life chemicals such as phthalates (DBP, BBzP, DEHP, and DINP) by measuring their urinary metabolites and examined the association between phthalate metabolite levels and health outcomes such as subjective symptoms, asthma, and allergic symptoms.

### Rationale of This Study

This study was guided by 5 key considerations. First, it aimed to identify the prevalence of health outcomes among e-waste workers. Second, it aimed to determine occupational and environmental factors associated with respiratory symptoms such as asthma and allergies. Third, it conducted medical examinations, vision tests, and biomonitoring of blood and urine samples. Fourth, it examined short half-life chemical exposure and the health burden of e-waste workers. Finally, the study aimed to provide evidence-based recommendations for safer practices, awareness, and reduction of occupational exposure.

### Hypothesis

We hypothesized that workers involved in informal e-waste processing in Vietnam who inadequately or inconsistently used PPE would have a higher prevalence of health issues. Furthermore, workers with a higher frequency of e-waste handling are expected to have higher levels of phthalate exposure due to direct contact and proximity to the e-waste environment. Finally, high phthalate exposure is hypothesized to be associated with subjective symptoms, asthma, and allergies.

## Methods

### Study Design, Study Site, and Participant Recruitment

This study followed a cross-sectional design to assess the health conditions and chemical exposure among informal e-waste workers. The selected site was Bui village in Cam Xa Commune, My Hao District, Hung Yen Province, which is a well-known informal e-waste processing area in northern Vietnam. Before the survey, we visited potential villages with informal e-waste processing communities. Other villages were excluded because e-waste activities had ceased or only a limited number of workers remained. Therefore, Bui village was selected as the study site due to the presence of an active e-waste processing community for evaluating occupational exposure among informal e-waste workers. The study population comprised adult e-waste processing workers. The village has an estimated population of approximately 1480 residents, most of whom know one another. This facilitated identification and recruitment of e-waste workers through collaboration with local community leaders and public health nurses. Through village-wide announcements combined with direct outreach by local community leaders and public health nurses, we aimed to reach all potential residents who actively engaged in e-waste processing in the village, thereby minimizing selection bias. Participants were eligible for inclusion if they were engaged in e-waste processing, had worked at least once in the past 6 months, and were aged ≥16 years. Individuals were excluded if they were pregnant, aged <16 years, or not currently residing in Bui village.

Field surveys were conducted on March 10 and 11, 2025. Village-wide announcements were made to encourage participation. In addition, public health nurses personally contacted each worker.

### Data Collection

Trained field-workers visited e-waste processing sites and provided brief information about the study to generate interest. On the survey days (March 10 and 11, 2025), participants who visited the survey venue received a detailed explanation of the study, and written informed consent was obtained from those who agreed to participate.

### Questionnaire

A structured questionnaire was developed in Vietnamese. An English version was prepared to serve as a common reference. All questionnaires were administered by trained research staff and were collected electronically. Details of the questionnaire are presented in Multimedia Appendix 1.

The questionnaire collected information on demographic characteristics, such as sex, age, income, education, and dwelling characteristics. Occupational information was also collected, including years of e-waste processing, daily or weekly work time, work type, and use of protective safety equipment. This section was partly adapted from a modified version of the Occupational Physical Activity Questionnaire [24].

Respiratory and allergic symptoms were assessed using the European Community Respiratory Health Survey. Asthma or wheezing was considered present if the respondent answered “yes” to the question, “Have you had wheezing or whistling in the chest in the past 12 months?” Allergic rhinoconjunctivitis was defined as positive when the participant reported nasal symptoms (sneezing, a runny nose, or nasal congestion not caused by a cold) in the past 12 months, accompanied by itchy and watery eyes. Eczema was defined as positive if the participant reported having an itchy rash lasting 6 months or more, which was present in the past 12 months and affected typical flexural areas, such as the elbows, knees, ankles, buttocks, neck, ears, or around the eyes.

Subjective symptoms related to the work environment were defined using the sick building syndrome (SBS) MM040EA questionnaire [25], with specific attention to symptoms that occurred at least once per week and which the respondents indicated were related to the work environment. These symptoms were categorized into 3 groups: general symptoms, including fatigue, feeling heavy headed, headache, nausea or dizziness, and difficulty concentrating; mucosal symptoms, including itching, burning, or irritation of the eyes, a stuffy or runny nose, nosebleeds, hoarse or dry throat, and cough; and dermal symptoms, including dry or flushed facial skin, scaling or itching of the scalp or ears, dry hands, itching, and red skin.

Finally, dietary habits were assessed through a food frequency questionnaire covering frequency of eating vegetables, rice, and meat.

### Blood and Urine Sample Collection and Measurement

Certified nurses collected 3 blood samples (2.0 mL each) from each participant using butterfly needles: 2 samples were collected in ethylenediaminetetraacetic acid (EDTA) anticoagulant tubes, and one sample was collected in a heparin tube. One EDTA tube was used to measure red blood cell count, white blood cell count, and hemoglobin levels, and the second EDTA tube was used to measure hemoglobin A_1c_ (HbA_1c_) levels. A heparin anticoagulant tube was used for the biochemical analysis of total cholesterol, high-density lipoprotein, low-density lipoprotein, uric acid, blood urea nitrogen, blood creatinine, and triglycerides.

The research team also collected spot urine samples in a 4-mL urine collection tube. Urine samples were assessed for pH, leucocytes, nitrite, blood, protein, glucose, ascorbic acid, and ketones. Levels of urobilinogen, urinary creatinine, and albumin were also measured. Two 3 mL aliquots of the urine samples were stored in amber glass bottles at −20 °C for phthalate analysis. At the end of each survey day, all the samples were transported to the Vietnam National Institute of Occupational and Environmental Health (NIOEH) laboratory in cooler boxes to ensure proper temperature control.

We selected 4 phthalates (DBP, BBzP, DEHP, and DINP) due to their widespread use as plasticizers in plastic materials and electronic products and their potential release during e-waste processing activities [1,16]. Ten urinary metabolites of these phthalates were measured as biomarkers of internal exposure. By November 2025, we completed the measurement of phthalate metabolites: monoisobutyl phthalate, mono-n-butyl phthalate, monobenzyl phthalate, mono (2-ethylhexyl) phthalate, mono (2-ethyl-5-oxohexyl) phthalate, mono (2-ethyl-5-hydroxyhexyl) phthalate, mono (2-ethyl-5-carboxypentyl) phthalate, monoisononyl phthalate, mono-hydroxy-isononyl phthalate, and mono-carboxy-isononyl phthalate. Pretreated urine samples were analyzed for phthalate metabolites using liquid chromatography-tandem mass spectrometry on an Agilent 1290 Infinity system coupled to a QTRAP 3200 mass spectrometer Sciex system (Sciex). Details of urine pretreatment can be found elsewhere [26]. Quality assurance and quality control procedures for phthalate metabolite measurement included determination of the limit of detection (LOD) based on 7 replicate measurements for each metabolite. Recovery rates of internal standards and native analytes were assessed using 7 replicates, ranging from 99% to 119% and 73% to 95%, respectively. A 10-point calibration curve (0-20 ng/mL) was used, and linearity was confirmed with *R*^2^>0.995 across all analytes. Water blank subtraction was applied to correct for background contamination. Intraday and interday precision were assessed using the 5 ng/mL standard across 4 measurement days. Samples with outlier or inconsistent values were reprepared or reanalyzed when necessary. A summary of quality assurance and quality control results is presented in Table S1 in Multimedia Appendix 1.

### Airway Inflammation Test

Fractional nitric oxide was measured using the single-breath online method and expressed as gas phase concentrations (parts per billion) following the 2025 American Thoracic Society and European Respiratory Society guidelines [27]. Measurements were performed using a NIOX VERO device calibrated according to the manufacturer’s instructions prior to data collection. Instructions in Vietnamese, such as to inhale to total lung capacity and exhale for 10 seconds at a constant flow rate, were provided to participants. Values were recorded only when acceptable flow criteria were met.

### Body Composition

The participants’ body composition, including height, weight, and waist circumference, was measured by trained survey staff. Body fat percentage and muscle mass were assessed using a Tanita MC-780A body composition analyzer (Tanita Co. Ltd).

### Data Management and Quality Assurance

The questionnaires were digitally programmed and accessed by trained research staff through a secure and designated link. The data were collected using handheld tablets. The responses were reviewed in real time by the research team and downloaded at the end of the survey. Once the data were cleaned and merged with the blood and urine test results and the phthalate exposure results, the dataset, together with the raw data, was stored on a password-protected solid-state drive at the NIOEH. Anonymized data will be made available to researchers upon request, subject to approval by the principal investigator at Hokkaido University and a designated representative from the NIOEH.

### Statistical Analysis

Data are presented as counts and percentages, means and SDs, or medians and IQRs. The distribution of urinary phthalate metabolite concentrations will be assessed for normality, and in the case of skewness, values were natural log-transformed for analysis. Concentrations below the LOD will be imputed as LOD/√2 values, and all concentrations will be adjusted for specific gravity to account for variations in urine dilution. The exposure variables comprised the concentrations of 10 phthalate metabolites. To address potential multicollinearity among these metabolites, we first assessed their interrelationships using Spearman correlation coefficients. The metabolites with the same parent phthalate compound are expected to demonstrate high correlation; therefore, we will use molar sums such as ∑DBP, ∑BBzP, ∑DEHP, and ∑DINP to represent cumulative exposure and to reduce multiple comparisons. The primary outcome variables include SBS, asthma, and allergies. The prevalence of health outcomes will be examined using the chi-square test or Student *t* test (2-tailed), depending on the distribution and type of the variables. Confounders will be identified based on evidence from previous literature and informed by the directed acyclic graph framework [28]. Multivariate logistic regression models will be used to determine the associations between exposure and binary health outcomes. The results are presented as odds ratios with 95% CIs and adjusted for potential confounding factors. Multiple linear regression will be used for continuous outcomes. To prevent overfitting and maintain model stability, categorical variables with sparse responses will be combined into logically similar groups. For instance, sparse working-day-per-week categories (eg, <1 day per week and 2-4 days per week) will be combined. To account for multiple comparisons across phthalate metabolites and outcomes, the Benjamini-Hochberg false discovery rate (FDR) procedure will be applied, and FDR-adjusted *P* values will be reported [29]. Missing covariate data will be handled using multiple imputation by chained equation [30]. Additional sensitivity analyses will include the exclusion of extreme phthalate metabolite concentrations. The significance level was set at *P*<.05. Statistical analyses were conducted using R (R Foundation for Statistical Computing), JMP (SAS Institute), and SPSS (IBM Corp).

### Ethical Considerations

Before conducting the survey, ethics approval was obtained from the institutional review board of the NIOEH; WHO Collaborating Centre (VTN-03); Ministry of Health, Vietnam; and the Faculty of Health Sciences, Hokkaido University, Japan (23-114). After providing a detailed explanation of the study, we obtained written informed consent from all participants.

## Results

As of March 11, 2025, a total of 253 adult e-waste processors have been enrolled in this study, representing all eligible active workers in the village. The following results represent the preliminary descriptive findings intended to demonstrate the feasibility of the study protocol and provide an initial characterization of the study population. The demographic and e-waste work–related characteristics of the participants are presented in Table 1. Most of the participants were female (163/253, 64.4%). Participants’ ages ranged between 16 and 83 years, with an average age of 52.5 (SD 14.1) years. Most participants had secondary school education. For 23.3% (60/253) of the participants, the monthly household income was <US $150 (3.7 million VNĐ; VNĐ 1,000,000=US $40 as of July 31, 2026). Among the participants, 86.1% (218/253) were employed and 13.8% (35/253) owned an e-waste workshop. More than 70% (181/253, 71.5%) of the respondents reported e-waste recycling as their main source of income, whereas others reported farming or plastic recycling as their primary income. Approximately 60% (156/253, 61.6%) of the workshops were indoors, whereas the rest were outdoors. Of the 253 participants, 62.0% (n=157) worked on e-waste processing for 5 to 8 hours per day, 15% (n=38) worked for less than 5 hours per day, and 22.9% (n=58) reported working for more than 8 hours per day. Overall, 94.8% (240/253) of the participants reported using protective equipment, such as dust masks, gloves, eye protection, and protective shoes, while working on e-waste processing.

**Table 1:** Demographic and electronic waste work–related characteristics of study participants (N=253).

Variables				Participants, n (%)
**Demographics**				
	**Sex**			
		Male	90 (35.5)	
		Female	163 (64.4)	
	**Age (years)**			
		Range	16-83	
		Average (SD)	52.5 (14.1)	
	**Highest level of education**			
		Never	8 (3.1)	
		Primary	54 (21)	
		Secondary	142 (56.1)	
		High school	42 (16.6)	
		College and above	7 (2.8)	
	**Monthly household income (VNĐ,** **million;** **equivalent to US $)**			
		<3.7 (<150)	60 (23.3)	
		3.7-7.5 (151-300)	56 (22.1)	
		7.5-11.3 (301-449)	49 (19.4)	
		11.3-15 (450-599)	27 (10.5)	
		>15 (>600)	47 (18.6)	
		Prefer not to answer	14 (5.4)	
**Electronic workshop (e-waste^a^)–related characteristics**				
	**E-waste workshop**			
		Owner	35 (13.8)	
		Employed	218 (86.1)	
	**Main source of income**			
		E-waste recycling	181 (71.5)	
		Other recycling	72 (28.4)	
	**E-waste workshop location**			
		Indoor	156 (61.6)	
		Outdoor	97 (38.3)	
	**Working hours per day**			
		<5	38 (15)	
		5-8	157 (62)	
		>8	58 (22.9)	
	**Use of protective equipment**			
		Yes	240 (94.8)	
		No	13 (5.1)	

^a^e-waste: electronic waste.

## Discussion

### Preliminary Findings

In this study, a survey was successfully conducted with 253 e-waste workers. The preliminary findings in this study provide initial insights into the demographic characteristics, potential gender-specific and occupation-related health outcomes, and lifestyle factors of adults in e-waste processing villages. The predominance of female workers and older adults raises concerns about the potential sex- and age-specific health concerns of occupational exposure in informal e-waste processing settings. The recycling processes in these settings are almost entirely manual. Many tasks require careful and meticulous work that is considered more suitable for women, such as waste collection, sorting, cleaning, and the separation of recyclable materials. In contrast, male workers are more often involved in physically demanding tasks such as lifting, carrying, and loading goods. The tasks primarily performed by women are also those associated with higher exposure to occupational hazards and harmful agents that may adversely affect health [31]. Many previous studies focused primarily on male workers; research in Vietnam has begun to draw attention to female e-waste workers, highlighting their exposure to pollutants and their related health outcomes [10,32]. For instance, a positive association between serum polychlorinated biphenyls levels and thyroid hormones was reported among women engaged in e-waste processing in northern Vietnam [10].

Notably, the participants’ ages ranged from 16 to 83 years, with a mean age of 52.5 (SD 14.1) years. This could indicate that many participants may have been engaged in work for extended periods. Educational attainment was mostly at the secondary school level, which may limit awareness of occupational hazards [7]. These demographic characteristics should be considered when examining the association between occupational exposure and health. Moreover, the study found that e-waste processing is the main source of income for over 70% of participants and nearly one-quarter reported a monthly household income of less than US $150 (<3.7 million VNĐ). These findings reflect the economic vulnerability of these communities, which are categorized as low-income workers by the Vietnamese government under decree number 30/2025/NĐ-CP [33]. Despite the recognized health burden associated with e-waste processing, low-income workers are likely to continue working as a means of livelihood. The reliance on e-waste recycling as a primary source of income highlights the socioeconomic context in which occupational exposure occurs. Although a high proportion of the participants reported using some form of PPE, further investigation is needed to understand the type of PPE used, consistency of its use, and its effectiveness in reducing occupational exposure and adverse health outcomes. However, these preliminary findings should be interpreted cautiously and will be further contextualized in future research using data from the Vietnamese population, if available, or from a similar population.

### Expected Outcomes

This study is expected to provide evidence on the health status, medical examination findings, occupational profiles, and dietary and lifestyle characteristics of informal e-waste workers in Vietnam. It will also examine their exposure levels to phthalates. While previous studies in Vietnam have primarily focused on exposure to heavy metals and POPs, this protocol addresses an important research gap by examining exposure to nonpersistent chemicals such as phthalates. By addressing this gap, this study is expected to provide new evidence on the health status of e-waste workers. Additionally, it is expected to examine the association between phthalate exposure and health status, including respiratory and allergic symptoms as well as laboratory blood test results. Furthermore, this study validates the protocol for measuring phthalate metabolites at the NIOEH. This achievement underscores the technical capacity of the NIOEH laboratory.

Currently, phthalate metabolite measurements have been completed, and data cleaning is underway in preparation for statistical analyses examining the associations between health outcomes and demographic, occupational, dietary, and lifestyle factors, as well as phthalate exposure levels.

### Strengths and Limitations

This protocol has notable strengths. First, the general health assessment includes a medical examination (blood pressure and heart rate); anthropometric measurements (height, weight, body fat percentage, muscle mass, and waist circumference); vision tests; and laboratory analysis of urine (pH, leucocytes, nitrite, blood, protein, glucose, ascorbic acid, ketones, urobilinogen, urinary creatinine, and albumin, in addition to phthalate metabolites) and blood (red blood cells, white blood cells, hemoglobin, HbA_1c_, triglycerides, cholesterol, low-density lipoprotein, high-density lipoprotein, uric acid, and creatinine). Together, these measurements provide a holistic evaluation of the health status of e-waste workers. Second, by measuring short half-life chemicals, such as phthalates, this study addresses an important research gap that has rarely been investigated in informal e-waste processing settings in Vietnam. Third, the use of standardized methods, including structured questionnaires and validated laboratory analyses, ensured the reliability of the results. Finally, ethical oversight from both Vietnamese and Japanese institutional review boards, combined with informed consent from the participants, strengthened the transparency and ethical rigor of the study.

However, this study has some limitations. Although recruitment was conducted through village-wide announcement and outreach by local community leaders and public nurses to reach all eligible e-waste workers, selection bias cannot be entirely excluded, as some eligible workers may have been unavailable on the survey days or declined to participate. This cross-sectional design limits the causal inference between phthalate exposure and health outcomes. Respiratory health outcomes and subjective symptoms were based on self-reported data, which may have been subject to a recall bias. A notable limitation is the reliance on a single measurement of urinary phthalate metabolite levels. Owing to the short half-lives of these compounds, a single measurement may not fully capture the variations in exposure over time. This could potentially lead to exposure misclassifications. Future studies would benefit from collecting multiple samples over extended periods to better characterize exposure. Additionally, this study did not include environmental measurements, such as air or dust. Instead, biomonitoring was prioritized to assess internal exposure, specifically to address the limited biological data in Vietnamese e-waste processing research. However, we acknowledge that the absence of environmental data limits our ability to attribute exposure to potential sources. Furthermore, the lack of a nonexposed comparison population limits interpretation of whether the associations between phthalate exposure and outcomes differ from those in the general population. Therefore, findings should be interpreted cautiously. We considered it important to first clarify the living conditions and realities of the people residing in the area; however, including an appropriate population comparison will be important to further evaluate the associations between occupational exposure and health outcomes in future studies. Finally, although all eligible, active adult e-waste workers in the study village were included, the findings may not be generalizable or applicable to other regions or formal recycling settings.

### Dissemination Plan

The findings of this study will be shared through multilevel dissemination. At the community level, participants were provided with individual clinical blood results, ensuring full confidentiality. A summary of the findings will be shared through local seminars targeting the community, key stakeholders, and relevant government authorities to foster local occupational health awareness. At the scientific level, the results will be submitted for publication in domestic and international peer-reviewed journals supplemented by oral or poster presentations at relevant domestic and international conferences.

## Appendix

Multimedia Appendix 1 Survey questionnaires and summary of quality assurance and quality control results for urinary phthalate metabolites measurement in study participants.

## Abbreviations

BBzP: butyl benzyl phthalate
CVD: cardiovascular disease
DBP: dibutyl phthalate
DEHP: di-(2-ethylhexyl) phthalate
DINP: diisononyl phthalate
EDTA: ethylenediaminetetraacetic acid
e-waste: electronic waste
FDR: false discovery rate
HbA1c: hemoglobin A1c
LOD: limit of detection
NIOEH: National Institute of Occupational and Environmental Health
POP: persistent organic pollutant
PPE: personal protective equipment
SBS: sick building syndrome

## Data Availability

The data are not publicly available due to ethical restrictions and privacy protection.
