# Supplementary material for: Investigation of Exposure to Plasticizers Among Electronic Waste Recycling Workers and Their Health: Protocol for a Cross-Sectional Study
Source: JMIR Res Protoc. 2026 Aug 18;15:e96702. doi: 10.2196/96702 (PMC13535303; doi:10.2196/96702)
Supplement: Supplementary file 1 [file resprot_v15i1e96702_app1.docx]

**Supplementary Material**

**Bộ câu hỏi phỏng vấn EXCELLENT/ EXCELLENT Survey Questionnaire**

**Section 0: Administrative**

1. Ngày phỏng vấn/ Date of the survey
2. Mã số / ID
3. Người phỏng vấn/ Interviewer

**Section 1: Phần A: Thông tin cá nhân & nhân khẩu học /Demographics and Personal Information Đủ điều kiện tham gia phỏng vấn (≥16 tuổi, sống tại Thôn Bùi, không mang thai) Description: Eligibility status (≥16 years old, Bui Village resident, non-pregnant)**

1. Giới tính/	Sex

1) Nam/	Male ( chuyển đến câu
2) Nữ giới/ Female

1.1. Nếu là nữ, bạn có đang mai thai không?/ If "1" is "Female", please specify if you are pregnant

1) Có Yes / ( Dừng vì không đủ điều kiện tham gia nghiên cứu)
2) Không No
3) Không muốn trả lời/ Prefer not to answer

2. Năm sinh / Year of Birth

3. Đã sinh sống ở thôn Bùi được mấy năm (năm)? Duration of residence in Bui Village (years)

4. Tình trạng hôn nhân? Relationship status

1) Đã kết hôn	/ Married
2) Độc thân / Single
3) Ly thân/Ly hôn/Goá Divorced/Separated/Widow

5. Trình độ học vấn	Education level

1) Không, chưa từng học /None/Never
2) Tiểu học /Primary
3) Trung học cơ sở / Secondary
4) Trung học phổ thông /High School
5) College and above / Cao đẳng và đại học trở lên

6. Tổng thu nhập hàng tháng của hộ gia đình / Household monthly income

1. < 3,7 triệu VNĐ (<$ 150 ~ <3.7M VND / < 150 USD)

2. 3,7 - 7,5 triệu VNĐ ($151-300 ~ 3.7 M - 7.5 M VND / 151-300 USD)

3. 7,5 - 11,3 triệu VNĐ ($301-449 ~ 7.5 M-11.3 M VND / 301-449 USD)

4. 11,3 - 15 triệu VNĐ ($450-599 ~ 11.3-15 M VND / 450 - 599 USD)

5. >15 triệu VNĐ (>$600 ~ >15 m VND / > 600 USD)

6. Không muốn trả lời Prefer not to answer /

7. Anh/Chị có hút thuốc không? Do you smoke?

1. Có/ Yes

2. Không/ No

3. Trước đây có hút nhưng giờ đã bỏ thuốc / Previously smoked/quit

8. Anh/Chị có uống rượu không?/ Do you drink alcohol?

1. Không bao giờ / Never

2. Có, thỉnh thoảng (1-2 lần/tuần)/ Yes (occasionally 1-2/week)

3. Có, vài lần 1 tuần (3-5 lần/tuần)/ Yes (few times 3-5/week)

4. Có, hàng ngày (≥6 lần/tuần)/ Yes every day ((≥6/week)

**Section 2: Phần B. Hoạt động công việc (Bộ câu hỏi về hoạt động thể chất nghề nghiệp sửa đổi (OPAQ)/ Work activity (modified Occupational Physical Activity Questionnaire (OPAQ)**

**Sau đây, chúng tôi sẽ hỏi Anh/Chị một số câu hỏi liên quan đến công việc của Anh/Chị.**

9. Vai trò của Anh/chị tại xưởng tái chế? The recycling workshop

1. Chủ sở hữu/ Owner

2. Nhân viên/Công nhân/ Employed

10. Nơi ở của Anh/Chị có tách biệt với xưởng tái chế không?/ Is your residence separate from the recycling workshop?

1. Có / Yes

2. Không/ No

11. Nguồn thu nhập chính của Anh/Chị là từ? Main source of income

1. Tái chế chất thải điện tử/ E-waste recycling

2. Tái chế chất thải khác/ Other recycling

3. Khác (ghi rõ) / Other (Please specify)

Other:

12. Anh/Chị thực hiện tái chế chất thải ở đâu?/ Where do you do your recycling activity?

1. Trong nhà/Khu vực kín (xưởng) / Indoor/ enclosed space (Workshop)

2. Ngoài trời/Không gian mở/ Outdoor/ Open space

12.1 Khu vực làm việc thường xuyên của Anh/chị có hệ thống thông gió không? / Does your work area have ventilation system?

1. Có / Yes

2. Không/ No

12.2 Khu vực làm việc thường xuyên của Anh/chị có đủ ánh sáng không? Does your work area have good lighting?

1. Có / Yes

2. Không/ No

13. Anh/Chị đã làm nghề tái chế chất thải điện tử được mấy năm rồi (năm) ? How long have you been working in e-waste recycling (years)?

14. Mỗi ngày, Anh/Chị làm việc mấy giờ?/ How many hours do you work per day?

1. Dưới 5 giờ / Less than 5 hours

2. 5-8 giờ /5-8 hours

3. Trên 8 giờ / More than 8 hours

15. Anh/Chị làm việc bao nhiêu ngày mỗi tuần?/ How many days do you work in a week?

1. Hàng ngày (6-7 ngày) / Everyday (6-7 days)

2. Gần như hàng ngày (5 ngày)/ Almost every day (5 days)

3. 2-4 ngày /2-4 days

4. Một ngày hoặc ít hơn/ One day or less

5. Không biết/không chắc chắn/ Don’t know/Not sure

16. Anh/chị tham gia công đoạn nào trong việc tái chế chất thải điện tử? What is your role in e-waste recycling? (có thể chọn nhiều đáp án)/ (multiple choice possible)

1. Người trực tiếp tái chế (trích xuất/phân tách/sản xuất vật liệu hữu ích từ chất thải điện tử) / Recycler (extract/produce useful materials from e-waste)

2. Thu gom / Collector

6. Nấu chảy/ Smelting

7. Đốt/ Burning

8. Khác (ghi rõ)/ Other (please Specify)

Other:

17. Anh/Chị tái chế loại chất thải điện tử nào? (có thể chọn nhiều đáp án)

What type of e-waste do you handle? (multiple choice possible)*

1. Tivi/ TV

2. Tủ lạnh/ Refrigerator

3. Máy giặt / Washing Machine

4. Máy điều hoà/ Air conditioner

5. Máy tính/laptop/ PC/laptop computer

6. Khác (điện thoại di động, máy ảnh, máy nghe nhạc, máy in)/ Others (cell phone, camera, music player, printer, etc)/ Ghi rõ

18. Anh/Chị có sử dụng phương tiện bảo vệ cá nhân khi tái chế không?/ Do you use any protective equipment while recycling? *

1. Có / Yes

2. Không/ No / ( chuyển đến 18.2)

18.1. Nếu “Có” Anh/Chị sử dụng phương tiện bảo vệ cá nhân nào trong khi làm việc? (Có thể chọn nhiều)

If 18 is "Yes", what protective equipment do you use while working? (multiple choice possible)

1. Khẩu trang chống bụi / Dust mask

2. Găng tay/ Glove

3. Kính bảo hộ / Eye protection/Goggles

4. Thiết bị bảo vệ tai (nút tai, chụp tai giảm ồn) / Hearing protection (ear plug/earmuffs for noise reduction)

5. Giầy bảo hộ / Ủng mũi thép / Protective shoes/ Steel-toe boots

18.2. Nếu câu trả lời cho câu hỏi 18 là "Không", nêu rõ lý do Anh/Chị không sử dụng phương tiện bảo vệ cá nhân?/ If 18 is "No", what is the reason you do not use protective equipment?

19. Anh/Chị có làm việc ở tư thế đứng ≥4 giờ/ngày không?

Do you work standing for ≥4 hours/day? *

1. Không bao giờ / Never

2. Thỉnh thoảng (1-2 ngày)/ Sometimes (1-2 Days)

3. Thường xuyên (3-5 ngày)/ Often (3-5 Days)

4. Hầu như hàng ngày (6-7 ngày)/ Almost always (6-7 Days)

20. Anh/Chị có làm việc ở tư thế ngồi ≥4 giờ/ngày không?*

Do you work sitting for ≥4 hours/day? *

1. Không bao giờ / Never

2. Thỉnh thoảng (1-2 ngày)/ Sometimes (1-2 Days)

3. Thường xuyên (3-5 ngày)/ Often (3-5 Days)

4. Hầu như hàng ngày (6-7 ngày)/ Almost always (6-7 Days)

21. Anh/Chị có làm việc ở tư thế quỳ/ ngồi xổm ≥4 giờ/ngày không?*

Do you work squatting/kneeling for ≥4 hours/day? *

1. Không bao giờ / Never

2. Thỉnh thoảng (1-2 ngày)/ Sometimes (1-2 Days)

3. Thường xuyên (3-5 ngày)/ Often (3-5 Days)

4. Hầu như hàng ngày (6-7 ngày)/ Almost always (6-7 Days)

22. Anh/Chị có đẩy/ kéo/ nâng hoặc mang vác các vật nặng (hơn 5kg) không?*

Do you push/pull/lift or carry heavy items (more than 5kg)? *

1. Không bao giờ / Never

2. Thỉnh thoảng (1-2 ngày)/ Sometimes (1-2 Days)

3. Thường xuyên (3-5 ngày)/ Often (3-5 Days)

4. Hầu như hàng ngày (6-7 ngày)/ Almost always (6-7 Days)

23. Anh/Chị có làm việc với các dụng cụ/ máy móc rung ≥4 giờ/ngày không?*

Do you work at vibrating tools/machines for ≥4 hours/day?*

1. Không bao giờ / Never

2. Thỉnh thoảng (1-2 ngày)/ Sometimes (1-2 Days)

3. Thường xuyên (3-5 ngày)/ Often (3-5 Days)

4. Hầu như hàng ngày (6-7 ngày)/ Almost always (6-7 Days)

23. 1. Anh/Chị có thường xuyên tiếp xúc với tiếng ồn lớn/ máy móc tại nơi làm việc không?/ How often are you exposed to loud noise/machines at work? *

1. Không bao giờ/ Never

2. Hầu như không bao giờ (2-3 lần/năm)/ Almost never (2-3 times a year)

3. Thỉnh thoảng (ít nhất 1 lần/tháng)/ Sometimes (at least once a month)

4. Khá thường xuyên (≥2lần/tháng)/ Fairly often (≥ 2 times a month)

5. Rất thường xuyên (≥ 5 lần/tháng)/ Very often (≥ 5 times a week)

24. Anh/Chị có đưa các thành viên nhỏ tuổi trong gia đình (con cái) đến khu vực tái chế chất thải điện tử không? /Do you bring your young family member/s (child) to your e-waste workplace? *

1. Không/ No / ( Chuyển đến Phần C)

2. Có/ Yes

24.1. Nếu câu trả lời cho câu hỏi 24 là "Có", xin cho biết mối quan hệ và độ tuổi của thành viên đó trong gia đình ( Ghi rõ ) / If 24 response is “Yes”, please state the relationship with you and the age of the family member

After section 4

Section 5 of 10

Phần C. Tự đánh giá sức khoẻ tổng quát và câu hỏi về Hội chứng bệnh nhà kín (Sick Building Syndrome - SBS) / Self-report overall health and Sick Building Syndrome (SBS) questions

Description: We would like to ask you some questions about your health.

25. Anh/Chị đánh giá tình trạng sức khỏe tổng quát của mình như thế nào? / How do you define your overall health? *

1. Tuyệt vời/Xuất sắc Excellent

2. Very good / Rất tốt Very good

3. Tốt/ Good

4. Khá tốt/ Fair

5. Kém/ Poor

26. Anh/Chị đã từng bị thương khi tái chế chất thải chưa? Have you ever been injured while recycling? *

1. Có/ Yes

2. Không/ No / ( chuyển đến câu 27)

26.1 Nếu câu trả lời cho câu hỏi 26 là "Có", hãy mô tả cụ thể hơn loại chấn thương /thương tích.( Ghi rõ ) / If 26 response is "Yes" please describe the injury type

27. Anh/Chị có bị đau đầu khi thực hiện hoạt động tái chế chất thải không?*

Do you experience headaches during the recycling activity? *

1. Có/ Yes

2. Không/ No / ( chuyển đến câu 28)

27.1 Nếu câu trả lời cho câu hỏi 27 là "Có", hãy mô tả mức độ nghiêm trọng của cơn đau đầu/ If 27 response is "Yes" please describe the severity of the headache

1. Đau nhẹ/ Mild

2. Rất đau/ Very painful

28. Anh/Chị có bị đau lưng khi thực hiện hoạt động tái chế chất thải không?* Do you experience back pain during the recycling activity? *

1. Có/ Yes

2. Không/ No / ( chuyển đến câu 29)

28.1 Nếu câu trả lời cho câu hỏi 28 là "Có", hãy mô tả mức độ nghiêm trọng của cơn đau lưng. If 28 response is "Yes" please describe the severity of the backpain

1. Đau nhẹ/ Mild

2. Rất đau/ Very painful

29. Anh/Chị có gặp khó khăn khi nghe không / Do you have difficulties of hearing? *

1. Có/ Yes

3. Không/ No / ( chuyển đến câu 30)

29.1 Nếu câu trả lời cho câu hỏi 29 là "Có", thời điểm Anh/Chị thấy khó nghe là khi nào?/ If 29 response is “Yes” " please indicate the time you noticed the difficulties in hearing

1. Từ khi còn nhỏ/ Since childhood

2. Sau khi tham gia tái chế chất thải điện tử/ After starting e-waste recycling

29.2 Anh/Chị có bị ù tai (tiếng kêu trong tai) trong/ sau khi tiếp xúc với tiếng ồn lớn tại nơi tái chế chất thải điện tử không? Have you experienced tinnitus (ringing in the ears) during/after spending time in loud noise at e-waste work?

1. Không bao giờ/ Never

2. Hiếm khi (2-3 lần/năm)/ Seldome (2-3 times a year)

3. Thỉnh thoảng (ít nhất 1 lần/tháng)/ Sometimes (at least once a month)

4. Khá thường xuyên (≥ 2 lần/tháng)/ Faily ofter (≥ 2 times a month)

5. Rất thường xuyên (≥ 5 lần/tuần)/ Very often (≥ 5 times a week)

29.3 Anh/Chị đã từng được chẩn đoán bị mất/giảm thính lực chưa? Have you ever been diagnosed by a doctor with hearing loss?

1. Có/ Yes

2. Không/ No

30. Anh/Chị có bị đau tay/ cổ tay trong hoặc sau khi tái chế chất thải điện tử không?*

Do you have pain in your hands/wrists during/after e-waste work? *

1. Có/ Yes

2. Không/ No / ( chuyển đến câu 31)

30.1 Nếu câu trả lời cho câu hỏi 30 là "Có", hãy mô tả mức độ đau tay/ cổ tay/

If 30 response is “Yes”, Describe a scale of pain in hands/wrists

1. Đau nhẹ/ Mild

2. Rất đau/ Very painful

31. Anh/Chị có bị đau cơ do ngồi lâu ở một vị trí trong khi làm tái chế không?/ Do you feel muscle soreness from sitting in the same position during recycling activity? *

1. Có/ Yes

2. Không/ No / ( chuyển đến câu 32)

31.1 Nếu câu trả lời cho câu hỏi 31 là "Có", hãy mức độ đau cơ như thế nào/

If 31 response is “Yes”, Describe a scale of pain of muscle soreness

1. Mild / Đau nhẹ

2. Very painful / Rất đau

32. Anh/Chị có mắc bệnh mạn tính nào không? (Chọn nhiều đáp án)

Please tell us if you are suffering/diagnosed with any chronic diseases. (Multiple answer is possible) *

1. Không có / None / ( chuyển đến câu 34)

2. Huyết áp cao/ High blood pressure

3. Bệnh tiểu đường/ Diabetes mellitus

4. Hen suyễn/ Asthma

5. Đột quỵ/ Stroke

6. Bệnh về thận/ Kidney disease

7. Bệnh về gan/ Liver disease

8. Tăng huyết áp/ Hypertension /

9. Khác (ghi rõ)/ Other (please specify

Other:

33. Anh/Chị có đang dùng thuốc điều trị cho bất kỳ bệnh nào trong số các bệnh trên không? Are you currently on medication for any of the above diseases?

1. Có / Yes

2. Không/ No

34. Anh/Chị có gặp bất kỳ vấn đề sức khỏe nào khiến Anh/Chị phải nghỉ làm hoặc làm ít đi không?* Do you have any health problems that limit your work? *

1. Có / Yes

2. Không/ No

35. Anh/Chị có bị giảm cân không chủ ý không? / Have you suffered from unintentional weight loss? *

1. Có / Yes

2. Không/ No

After section 5

Section 6 of 10

C-1. Bộ câu hỏi về Hội chứng bệnh nhà kín (SBS)/ Sick Building Syndrome (SBS) questionnaire / Xin vui lòng cho biết, Anh/Chị có bất kỳ triệu chứng nào sau đây trong ba tháng qua không? (Xin vui lòng trả lời từng câu hỏi ngay cả khi Anh/Chị không có triệu chứng)

Description: Please tell us if you had any symptoms during the last three months. (Please answer every question even if you have not had any symptoms)

36.1 Mệt mỏi/ Fatigue *

1. Có, thường xuyên (hàng tuần)/ Yes, often (every week)

2. Có, thỉnh thoảng/ Yes, sometimes

3. Không, không bao giờ/ No, never

36.1.1 Nếu câu trả lời cho câu hỏi 36.1 là "Có", Anh/Chị có cho rằng nguyên nhân là do môi trường làm việc không? / If 36.1 response is “Yes”, do you believe that it is due to your work environment? *

1. Có / Yes

Không/ No

Không biết/ Don't know

36.2 Cảm thấy nặng đầu/ Feeling heavy- headed *

1. Có, thường xuyên (hàng tuần)/ Yes, often (every week)

2. Có, thỉnh thoảng/ Yes, sometimes

3. Không, không bao giờ/ No, never

36.2.1 Nếu câu trả lời cho câu 36.2 là "Có", Anh/Chị có cho rằng nguyên nhân là do môi trường làm việc không? / If 36.2 response is “Yes”, do you believe that it is due to your work environment?

1. Có / Yes

2. Không/ No

3. Không biết/ Don't know

36.3 Đau đầu/ Headache *

1. Có, thường xuyên (hàng tuần)/ Yes, often (every week)

2. Có, thỉnh thoảng/ Yes, sometimes

3. Không, không bao giờ/ No, never

36.3.1 Nếu câu trả lời cho câu hỏi 36.3 là "Có", Anh/Chị có cho rằng nguyên nhân là do môi trường làm việc không? / If 36.3 response is “Yes”, do you believe that it is due to your work environment?

1. Có / Yes

2. Không/ No

3. Không biết/ Don't know

36.4 Buồn nôn, chóng mặt/ Nausea/dizziness *

1. Có, thường xuyên (hàng tuần)/ Yes, often (every week

2. Có, thỉnh thoảng/ Yes, sometimes

3. Không, không bao giờ/ No, never

36.4.1 Nếu câu trả lời cho câu hỏi 36.4 là "Có", Anh/Chị có cho rằng nguyên nhân là do môi trường làm việc không? / If 36.4 response is “Yes”, do you believe that it is due to your work environment?

1. Có / Yes

2. Không/ No

3. Không biết/ Don't know

36.5 Khó tập trung/ Difficulties concentrating *

1. Có, thường xuyên (hàng tuần)/ Yes, often (every week)

2. Có, thỉnh thoảng/ Yes, sometimes

3. Không, không bao giờ/ No, never

36.5.1 Nếu câu trả lời cho câu hỏi 36.5 là "Có", Anh/Chị có cho rằng nguyên nhân là do môi trường làm việc không? / If 36.5 response is “Yes”, do you believe that it is due to your work environment?

1. Có / Yes

2. Không/ No

3. Không biết/ Don't know

36.6 Ngứa, nóng rát hoặc kích ứng mắt/ Itching, burning or irritation of the eyes *

1. Có, thường xuyên (hàng tuần)/ Yes, often (every week)

2. Có, thỉnh thoảng/ Yes, sometimes

3. Không, không bao giờ/ No, never

36.6.1 Nếu câu trả lời cho câu hỏi 36.6 là "Có", Anh/Chị có cho rằng nguyên nhân là do môi trường làm việc không? / If 36.6 response is “Yes”, do you believe that it is due to your work environment?

1. Có / Yes

2. Không/ No

3. Không biết/ Don't know

36.7 Mũi kích ứng, nghẹt hoạc chảy nước mũi/ Irritated, stuffy or runny nose *

1. Có, thường xuyên (hàng tuần)/ Yes, often (every week)

2. Có, thỉnh thoảng/ Yes, sometimes

3. Không, không bao giờ/ No, never

36.7.1 Nếu câu trả lời cho câu hỏi 36.7 là "Có", Anh/Chị có cho rằng nguyên nhân là do môi trường làm việc không? / If 36.7 response is “Yes”, do you believe that it is due to your work environment?

1. Có / Yes

2. Không/ No

3. Không biết/ Don't know

36.8 Chảy máu cam/ Nose-bleeding

*

1. Có, thường xuyên (hàng tuần)/ Yes, often (every week)

2. Có, thỉnh thoảng/ Yes, sometimes

3. Không, không bao giờ/ No, never

36.8.1 Nếu câu trả lời cho câu hỏi 36.8 là "Có", Anh/Chị có cho rằng nguyên nhân là do môi trường làm việc không? / If 36.8 response is “Yes”, do you believe that it is due to your work environment?

1. Có, thường xuyên (hàng tuần)/ Yes, often (every week)

2. Có, thỉnh thoảng/ Yes, sometimes

3. Không, không bao giờ/ No, never

36.9 Họng khàn khàn, khô / Hoarse, dry throat

*

1. Có, thường xuyên (hàng tuần)/ Yes, often (every week)

2. Có, thỉnh thoảng/ Yes, sometimes

3. Không, không bao giờ/ No, never

36.9.1 Nếu câu trả lời cho câu hỏi 36.9 là "Có", Anh/Chị có cho rằng nguyên nhân là do môi trường làm việc không? / If 36.9 response is “Yes”, do you believe that it is due to your work environment?

1. Có / Yes

2. Không/ No

3. Không biết/ Don't know

36.10 Ho/ Cough

*

1. Có, thường xuyên (hàng tuần)/ Yes, often (every week)

2. Có, thỉnh thoảng/ Yes, sometimes

3. Không, không bao giờ/ No, never

36.10.1 Nếu câu trả lời cho câu hỏi 36.10 là "Có", Anh/Chị có cho rằng nguyên nhân là do môi trường làm việc không? / If 36.10 response is “Yes”, do you believe that it is due to your work environment?

1. Có / Yes

2. Không/ No

3. Không biết/ Don't know

36.11 Da mặt khô hoặc ửng đỏ/ Dry or flushed facial skin

*

1. Có, thường xuyên (hàng tuần)/ Yes, often (every week)

2. Có, thỉnh thoảng/ Yes, sometimes

3. Không, không bao giờ/ No, never

36.11.1 Nếu câu trả lời cho câu hỏi 36.11 là "Có", Anh/Chị có cho rằng nguyên nhân là do môi trường làm việc không? / If 36.11 response is “Yes”, do you believe that it is due to your work environment?

1. Có / Yes

2. Không/ No

3. Không biết/ Don't know

36.12 Da đầu hoặc tai bong tróc/ngứa/ Scaling/itching scalp or ears

*

1. Có, thường xuyên (hàng tuần)/ Yes, often (every week)

2. Có, thỉnh thoảng/ Yes, sometimes

3. Không, không bao giờ/ No, never

36.12.1 Nếu câu trả lời cho câu hỏi 36.12 là "Có", Anh/Chị có cho rằng nguyên nhân là do môi trường làm việc không? / If 36.12 response is “Yes”, do you believe that it is due to your work environment?

1. Có / Yes

2. Không/ No

3. Không biết/ Don't know

36.13 Da tay khô, ngứa, mẩn đỏ / Hand dry, itching, redskin

*

1. Có, thường xuyên (hàng tuần)/ Yes, often (every week)

2. Có, thỉnh thoảng/ Yes, sometimes

3. Không, không bao giờ/ No, never

36.13.1 Nếu câu trả lời cho câu hỏi 36.13 là "Có", Anh/Chị có cho rằng nguyên nhân là do môi trường làm việc không? / If 36.13 response is “Yes”, do you believe that it is due to your work environment?

1. Có / Yes

2. Không/ No

3. Không biết/ Don't know

36.14 Bị/Cảm thấy căng thẳng/	Suffering from stress

*

1. Có, thường xuyên (hàng tuần)/ Yes, often (every week)

2. Có, thỉnh thoảng/ Yes, sometimes

3. Không, không bao giờ/ No, never

36.14.1 Nếu câu trả lời cho câu hỏi 36.14 là "Có", Anh/Chị có cho rằng nguyên nhân là do môi trường làm việc không? / If 36.14 response is “Yes”, do you believe that it is due to your work environment?

1. Có / Yes

2. Không/ No

3. Không biết/ Don't know

36.15 Dễ cáu kỉnh về những việc nhỏ/	Easily irritated about small matters

*

1. Có, thường xuyên (hàng tuần)/ Yes, often (every week)

2. Có, thỉnh thoảng/ Yes, sometimes

3. Không, không bao giờ/ No, never

36.15.1 Nếu câu trả lời cho câu hỏi 36.15 là "Có", Anh/Chị có cho rằng nguyên nhân là do môi trường làm việc không? If 36.15 response is “Yes”, do you believe that it is due to your work environment?

1. Có / Yes

2. Không/ No

3. Không biết/ Don't know

36.16 Khó ngủ/ Difficulties to sleep

*

1. Có, thường xuyên (hàng tuần)/ Yes, often (every week)

2. Có, thỉnh thoảng/ Yes, sometimes

3. Không, không bao giờ/ No, never

36.16.1 Nếu câu trả lời cho câu hỏi 36.16 là "Có", Anh/Chị có cho rằng nguyên nhân là do môi trường làm việc không? If 36.16 response is “Yes”, do you believe that it is due to your work environment?

1. Có / Yes

2. Không/ No

3. Không biết/ Don't know

36.17 Các triệu chứng khác (vui lòng mô tả ngắn gọn)/ Other (Please describe brief) Triệu chứng khác (ghi rõ)/ Other:________________________________________________

*

36.17.1 Nếu câu trả lời cho câu hỏi 36.17 là "Có", Anh/Chị có cho rằng nguyên nhân là do môi trường làm việc không?/ If 36.17 response is “Yes”, do you believe that it is due to your work environment?

1. Có / Yes

2. Không/ No

3. Không biết/ Don't know

After section 6

Section 7 of 10

C-2 Question about asthma / B C-2 Bộ câu hỏi về hen suyễn / Question about asthma

Description (optional)

37.1 Anh/Chị đã từng bị thở khò khè hoặc có tiếng huýt sáo trong ngực vào bất kỳ thời điểm nào trong 12 tháng qua không? / Have you ever had wheezing or whistling in the chest at any time in the past 12 months? If 37.1 response is “No” please skip to question 37.6)

*

1. Có/ Yes

2. Không/ No	( chuyển đến câu 37.6)

37.2 Anh/Chị đã bị thở khò khè hoặc có tiếng huýt sáo trong ngực trong 12 tháng qua không? Have you had wheezing or whistling in the chest in the past 12 months?

1. Có/ Yes

2. Không/ No	( chuyển đến câu 37.6)

37.3. Anh/Chị đã bị thở khò khè bao nhiêu lần trong 12 tháng qua? How many attacks of wheezing have you had in the past 12 months?

1. 1-3 lần / (1-3 times)

2. 4-12 lần/ (4-12 times)

3. Hơn 12 lần/ More than 12 times

37.4. Trong 12 tháng qua, giấc ngủ của Anh/Chị bị gián đoạn trung bình bao nhiêu lần do thở khò khè? In the past 12 months, how often, on average, has your sleep been disturbed due to wheezing?

1. Không bao giờ bị tỉnh giấc vì khó khè/ Never woken with wheezing

2. Một đêm mỗi tuần/ One night per week

3. Hơn một đêm mỗi tuần/ More than one nights per week

37.5 Trong 12 tháng qua, tình trạng thở khò khè có nghiêm trọng đến mức Anh/Chị chỉ nói được một hoặc hai từ giữa các nhịp thở không?/ In the past 12 months, has wheezing ever been severe enough to limit your speech to only one or two words at a time between breaths?

1. Có/ Yes

2. Không/ No

37.6 Anh/Chị đã từng được bác sĩ chẩn đoán là bị hen suyễn chưa?* Have you ever had asthma (diagnosed by doctor)?

*

1. Có/ Yes

2. Không/ No

37.7 Trong 12 tháng qua, Anh/Chị có bị tiếng thở khò khè ở lồng ngực trong hoặc sau khi tập thể dục không?* In the past 12 months, has your chest sounded wheezy during or after exercise?

*

1. Có/ Yes

2. Không/ No

37.8 Trong 12 tháng qua, Anh/Chị có bị ho khan vào ban đêm nhưng không phải là do cảm lạnh hoặc nhiễm trùng ngực không?* / In the past 12 months, have you had a dry cough at night, apart from a cough associated with a cold or chest infection?

*

1. Có/ Yes

2. Không/ No

After section 7

Section 8 of 10

C-3 Bộ câu hỏi về bệnh viêm mũi dị ứng/ Question about Allergic rhinitis / Tất cả các câu hỏi dưới đây đều về các triệu chứng xuất hiện khi Anh/Chị KHÔNG bị cảm lạnh hoặc cúm./

All questions are about symptoms that occur when you DO NOT have a cold or the flu / Tất cả các câu hỏi dưới đây đều về các triệu chứng xuất hiện khi Anh/Chị KHÔNG bị cảm lạnh hoặc cúm.

38.1 Anh/Chị đã từng gặp vấn đề với hắt hơi, sổ mũi, hoặc nghẹt mũi khi Anh/Chị KHÔNG bị cảm lạnh hoặc cúm không? / Have you ever had a problem with sneezing or a runny, or blocked nose when you DID NOT have a cold or the flu? If 38.1 response is “No” please skip to question 38.6)

*

1. Có/ Yes

2. Không/ No ( chuyển đến câu 38.6)

38.2 Trong 12 tháng qua, Anh/Chị đã bị hắt hơi, sổ mũi, hoặc nghẹt mũi khi Anh/Chị KHÔNG bị cảm lạnh hoặc cúm không?/ In the past 12 months, have you had a problem with sneezing or a runny, or blocked nose when you DID NOT have a cold or the flu? (If 38.2 response is “No” please skip to question 38.6) / Nếu câu trả lời cho câu hỏi 38.2 là "Không", xin vui lòng bỏ qua câu hỏi 38.6)

1. Có/ Yes

2. Không/ No ( chuyển đến câu 38.6)

38.3 Trong 12 tháng qua, vấn đề mũi này có kèm theo ngứa và chảy nước mắt không?/ In the past 12 months, has this nose problem been accompanied by itchy-watery eyes?

1. Có/ Yes

2. Không/ No

38.4 In which month of the past 12 months did this nose problem occur? (Please tick if any apply) /. Trong 12 tháng qua, vấn đề mũi này xảy ra vào tháng nào? (Xin vui lòng đánh dấu nếu có)

1. Tháng 1/ January

2. Tháng 2/ February

3. Tháng 3/ March

4. Tháng 4/ April

5. Tháng 5/ May

6. Tháng 6/ June

7. Tháng 7/ July

8. Tháng 8/ August

9. Tháng 9/ September

10. Tháng 10/ October

11. Tháng 11/ November

12. Tháng 12/ December

38.5 Trong 12 tháng qua, vấn đề mũi này đã ảnh hưởng đến hoạt động hàng ngày của Anh/Chị như thế nào? In the past 12 months, how much did this nose problem interfere with your daily activities?

1. Không ảnh hưởng/ Not at all

2. Ảnh hưởng ít/ A little

3. Ảnh hưởng trung bình/ A moderate amount

4. Ảnh hưởng nhiều/ A lot

38.6 Anh/Chị đã từng bị dị ứng phấn hoa chưa?/ Have you ever had hay fever?

*

1. Có/ Yes

2. Không/ No

After section 8

Section 9 of 10

C-4 Bộ câu hỏi về bệnh viêm da cơ địa (Eczema) / C-4 Question for eczema

Description (optional)

39.1 Anh/Chị đã từng bị phát ban ngứa liên tục trong ít nhất 06 tháng chưa? Have you ever had an itchy rash which was coming and going for at least six months? /

(Nếu câu trả lời cho câu hỏi 39.1 là "Không", xin vui lòng chuyển đến câu hỏi 39.6/ If 39.1 response is “No” please skip to question 39.6)

*

1. Có/ Yes

2. Không/ No ( chuyển đến câu 39.6)

39.2 Anh/Chị đã bị phát ban ngứa như thế này vào bất kỳ thời điểm nào trong trong 12 tháng qua không?/ Have you had this itchy rash at any time in the past 12 months? /

(Nếu câu trả lời cho câu hỏi 39.2 là "Không", xin vui lòng bỏ qua câu hỏi 39.4/ If 39.2 response is “No” please skip to question 39.4)

1. Có/ Yes

2. Không/ No ( chuyển đến câu 39.6)

39.3 Phát ban ngứa này có từng ảnh hưởng đến bất kỳ vị trí nào trên cơ thể Anh/Chị như: nếp gấp khuỷu tay, sau đầu gối, trước mắt cá chân, dưới mông hoặc quanh cổ, tai hoặc mắt không? / Has this itchy rash at any time affected any of the following places: The fold of the elbows, behind the knees, in front of the ankles, under the buttocks, or around the neck, ears or eyes?

1. Có/ Yes

2 . Không/ No

39.4	Phát ban ngứa này có tự khỏi hoàn toàn vào bất kỳ thời điểm nào trong 12 tháng qua không? / Has this rash cleaned completely at any time during the past 12 months?

1. Có/ Yes

2. Không/ No

39.5 Trong 12 tháng qua, phát ban ngứa này đã khiến Anh/Chị mất ngủ vào ban đêm trung bình bao nhiêu lần? / In the past 12 months, how often, on average, have you been kept awake at night by this itchy rash?

1. Không bao giờ trong 12 tháng qua/ Never in the past 12 months /

2. Một đêm mỗi tuần/One night per week

3. Nhiều hơn một đêm mỗi tuần/ More than one night per week

39.6 Anh/Chị đã từng bị bệnh viêm da cơ địa chưa? / Have you ever had eczema?

1. Có/ Yes

2. Không/ No

After section 9

Section 10 of 10

BỘ CÂU HỎI TẦN SUẤT TIÊU THỤ THỰC PHẨM (FFQ) /FOOD FREQUENCY QUESTIONNAIRE (FFQ) / C

họn một (01) phương án trả lời phù hợp nhất với Anh/Chị cho mỗi câu hỏi!/ Select best option for each question - indicate what you usually do!

Anh/Chị ăn rau (tươi, đông lạnh hay nấu chín) thường xuyên như thế nào?*/ How often do you eat vegetables (fresh, frozen, or cooked)?

*

1. Hai lần một ngày hoặc thường xuyên hơn/ Twice a day or more often

2. Một lần mỗi ngày/ Once a day

3. Một vài lần trong tuần/ A few times a week

4. Một lần mỗi tuần hoặc ít hơn/ Once a week or less

41. Anh/Chị ăn trái cây và/hoặc quả mọng (tươi, đông lạnh, bảo quản, nước ép, v.v.) thường xuyên như thế nào?*/ How often do you eat fruit and/or berries (fresh, frozen, preserved, juice, etc.)?

*

1. Hai lần một ngày hoặc thường xuyên hơn/ Twice a day or more often

2. Một lần mỗi ngày/ Once a day

3. Một vài lần trong tuần/ A few times a week

4. Một lần mỗi tuần hoặc ít hơn/ Once a week or less

42. Anh/Chị ăn các loại hạt (hạnh nhân, đậu phộng, hạt macca, hạt dẻ cười, hạt thông, hạt óc chó, hạt điều) thường xuyên như thế nào?*/ How often do you eat nuts (almonds, peanuts, hazelnuts, pistachio nuts, pine nuts, walnuts, cashews)?

*

1. Hai lần một ngày hoặc thường xuyên hơn/ Twice a day or more often

2. Một lần mỗi ngày/ Once a day

3. Một vài lần trong tuần/ A few times a week

4. Một lần mỗi tuần hoặc ít hơn/ Once a week or less

43. Anh/Chị ăn cá hoặc hải sản có vỏ thường xuyên như thế nào? * / How often do you eat fish or shellfish?

*

1. Ba lần mỗi tuần hoặc thường xuyên hơn/ Three times a week or more often

2. Hai lần mỗi tuần/ Twice a week

3. Một lần mỗi tuần/ Once a week or less

4. Một vài lần mỗi tháng hoặc ít hơn/ A few time a month or less

44. Anh/Chị ăn thịt đỏ (thịt bò hoặc thịt lợn, hoặc thịt thú rừng) thường xuyên như thế nào?/ How often do you eat red meat (beef or pork)?

*

1. Ba lần mỗi tuần hoặc thường xuyên hơn/ Three times a week or more often

2. Hai lần mỗi tuần/ Twice a week

3. Một lần mỗi tuần/ Once a week or less

4. Một vài lần mỗi tháng hoặc ít hơn/ A few time a month or less

45. Anh/Chị ăn thịt trắng (thịt gia cầm như thịt gà) thường xuyên như thế nào?*/ How often do you eat white meat (poultry e.g. chicken)?

*

1. Ba lần mỗi tuần hoặc thường xuyên hơn/ Three times a week or more often

2. Hai lần mỗi tuần/ Twice a week

3. Một lần mỗi tuần/ Once a week or less

4. Một vài lần mỗi tháng hoặc ít hơn/ A few time a month or less

46. Anh/Chị ăn bánh bao/bánh ngọt, sô cô la/kẹo, khoai tây chiên hoặc soda/nước trái cây thường xuyên như thế nào?*/ How often do you eat buns/cakes, chocolate/sweets, crisps or soda/juice?

*

1. Hai lần một ngày hoặc thường xuyên hơn/ Twice a day or more often

2. Một lần mỗi ngày/ Once a day

3. Một vài lần trong tuần/ A few times a week

4. Một lần mỗi tuần hoặc ít hơn/ Once a week or less

47. Anh/Chị ăn sáng thường xuyên như thế nào?*/ How often do you eat breakfast?

*

1. Hàng ngày/ Everyday

2. Hầu như hàng ngày/ Almost everyday

3. Một vài lần trong tuần/ A few times a week

4. Một lần mỗi tuần hoặc ít hơn/ Once a week or less

48. Anh/Chị ăn bánh mì thường xuyên như thế nào?/ How often do you eat pieces of bread/ “banh mi” ?

*

1. Hai lần một ngày hoặc thường xuyên hơn/ Twice a day or more often

2. Một lần mỗi ngày/ Once a day

3. Một vài lần trong tuần/ A few times a week

4. Một lần mỗi tuần hoặc ít hơn/ Once a week or less

49. Anh/Chị uống/ăn sữa, sữa chua thường xuyên như thế nào?*/ How often do you drink/eat milk, sour milk and/or yoghurt?

*

1. Hai lần một ngày hoặc thường xuyên hơn/ Twice a day or more often

2. Một lần mỗi ngày/ Once a day

3. Một vài lần trong tuần/ A few times a week

4. Một lần mỗi tuần hoặc ít hơn/ Once a week or less

50. Anh/Chị ăn cơm tẻ thường xuyên như thế nào? / How often do you eat rice?/

*

1. Hai lần một ngày hoặc thường xuyên hơn/ Twice a day or more often

2. Một lần mỗi ngày/ Once a day

3. Một vài lần trong tuần/ A few times a week

4. Một lần mỗi tuần hoặc ít hơn/ Once a week or less

51. Anh/Chị ăn cơm nếp (xôi) thường xuyên như thế nào?/ How often do you eat sticky rice?

*

1. Hai lần một ngày hoặc thường xuyên hơn/ Twice a day or more often

2. Một lần mỗi ngày/ Once a day

3. Một vài lần trong tuần/ A few times a week

4. Một lần mỗi tuần hoặc ít hơn/ Once a week or less

52. Anh/Chị ăn cháo thường xuyên như thế nào?/ How often do you eat porridge?

*

1. Hai lần một ngày hoặc thường xuyên hơn/ Twice a day or more often

2. Một lần mỗi ngày/ Once a day

3. Một vài lần trong tuần/ A few times a week

4. Một lần mỗi tuần hoặc ít hơn/ Once a week or less

53. Anh/Chị ăn mì/miến/phở thường xuyên như thế nào? How often do you eat Vermicelli/ Rice noodles/Pho?

*

1. Hai lần một ngày hoặc thường xuyên hơn/ Twice a day or more often

2. Một lần mỗi ngày/ Once a day

3. Một vài lần trong tuần/ A few times a week

4. Một lần mỗi tuần hoặc ít hơn/ Once a week or less

54. Anh/Chị cho biết loại dầu, mỡ nào thường dùng nhất?/ What type(s) of most commonly used oils and fats?

*

1. Dầu thực vật/ Vegetable oils

2. Mỡ động vật/ Animal fats

3. Khác, ghi rõ/ Other, please specify

Other:

55. Anh/Chị tự thấy thói quen ăn uống của mình như thế nào?/ How do you feel about your eating habits?/ (Nhiều lựa chọn)

*

1. Ăn chay / Vegetarianism

2. Ăn ít chất béo/ Eat less fat

3. Ăn đồ mặn/ Eat salty food

4. Không có gì đặc biệt/ Nothing special

5. Khác ghi rõ/ Other, please specify

Other:

56. Anh/Chị cho biết các bữa ăn của mình có đều không?/ Can you tell us whether your meals are regular?

Question Type

1. Đều đặn/ Regular

2. Không đều (thất thường)/ Irregular (erratic)

or

Thank you very much for your valuable time and contribution / Trân trọng cảm ơn Anh/Chị đã dành thời gian quý báu cho cuộc phỏng vấn!

Supplemental Table 1: Summary of quality assurance and quality control results

| Metabolites | R^2^ | IS  recovery % | CV % | Native recovery % | CV% | LOD (ng/mL) | Mean Inter-day RSD% | Mean Intra-day RSD% |
| --- | --- | --- | --- | --- | --- | --- | --- | --- |
| MiBP | 0.997 | 108 | 4.2 | 87 | 5.2 | 1.31 | 4.9 | 8.2 |
| MnBP | 0.997 | 111 | 6.5 | 93 | 6.0 | 2.26 | 8.6 | 9.9 |
| MBzP | 0.995 | 117 | 2.7 | 95 | 7.1 | 1.82 | 7.8 | 9.1 |
| MEHP | 0.996 | 119 | 3.8 | 90 | 4.0 | 0.27 | 10.6 | 7.8 |
| MEOHP | 0.995 | 114 | 3.9 | 73 | 4.7 | 0.15 | 3.0 | 5.9 |
| MEHHP | 0.998 | 99 | 6.2 | 78 | 5.3 | 0.73 | 6.0 | 7.0 |
| MECPP | 0.998 | 111 | 3.6 | 81 | 10.4 | 0.69 | 5.7 | 7.8 |
| MiNP | 0.996 | 116 | 4.6 | 85 | 7.5 | 1.67 | 11.6 | 6.8 |
| OH-MiNP | 0.999 | 116 | 2.0 | 93 | 2.2 | 0.43 | 4.7 | 4.3 |
| cx-MiNP | 0.999 | 112 | 5.5 | 84 | 6.4 | 0.43 | 6.1 | 6.5 |

R^2^: Coefficient of determination of the calibration curve linearity; IS (Internal standard) recovery %; native mean recovery %; CV: coefficient of variation; LOD (limit of detection); LOQ (limit of quantification); RSD (relative standard deviation). Inter-day precision was calculated as the RSD (%) of daily mean concentrations across 4 measurement days; intra-day precision was calculated as the mean RSD (%) of 4 measurement days at a concentration of 5 ng/mL.
